# 

**Published:** 1888-07-07

**Authors:** 


### CONTENTS.

**The General Practitioner's Indictment of the Hospitals** - 221

**Great Wit and Madness** - 222

**The Doctor's Pulpit.**—The Choice of a Health Resort - 223

**Hints for the Sick-room.** By M. M. Basil, M.A., M.B. - 224

**The Hospitals Association and Patients' Contributions** - 225

**Round about the Asylums.**—X. Lunatic Hospitals, Private and Pauper Asylums—Sussex County Asylum—Northampton County Asylum - 227

**Notes and News** - 228

**Everybody's Page** - 230

**Annotations.**—Summer Underclothing.—Wealth for Women, etc. 231

**Our First Hospital Saturday.** By A. W. Webster, Chairman of Workmen's Governors', Sunderland Infirmary - 232

**An Ishmaelite.** By Leslie Keith, Author of "The Chilcotes," "East and West," etc. - 233

**The Editor's Letter-box.**—Phrenology: A Protest—Thunderstorms and Lightning Accidents, etc. - 234

**St. Katherine's Hospital** - 235

**Amusements with Profit for all Ages.**—For Men and Women.—Children's Corner.—Puzzles, etc. - 236

**EXTRA SUPPLEMENT.—THE NURSING MIRROR.**

En Passant.—A Brave Nurse.—News from Sheffield.—A Nurse's Breach of Promise.—Actress and Nurse.—Diet of Nurses—The Nightingale Fund - liii
The National Pension Fund for Nurses - liv
Notes from America.—Nurses and Politics - liv
Everybody's Opinion.—Nurses and Patients.—The Pay of Private Nurses.—A Probationer's Protest.—Unpleasant Duties - lv
Notes and Queries.—Appointments - lvi
Examination Questions - lvi
Vacancies - lvi

